# Nebulized mRNA‐Encoded Antibodies Protect Hamsters from SARS‐CoV‐2 Infection

**DOI:** 10.1002/advs.202202771

**Published:** 2022-10-31

**Authors:** Daryll Vanover, Chiara Zurla, Hannah E. Peck, Nichole Orr‐Burks, Jae Yeon Joo, Jackelyn Murray, Nathan Holladay, Ryan A. Hobbs, Younghun Jung, Lorena C. S. Chaves, Laura Rotolo, Aaron W. Lifland, Alicia K. Olivier, Dapeng Li, Kevin O. Saunders, Gregory D. Sempowski, James E. Crowe, Barton F. Haynes, Eric R. Lafontaine, Robert J. Hogan, Philip J. Santangelo

**Affiliations:** ^1^ Wallace H. Coulter Department of Biomedical Engineering Emory University and Georgia Institute of Technology Atlanta GA 30322 USA; ^2^ Department of Infectious Diseases College of Veterinary Medicine University of Georgia Athens GA 30602 USA; ^3^ Department of Pathobiology and Population Medicine College of Veterinary Medicine Mississippi State University Starkville MS 39762 USA; ^4^ Duke Human Vaccine Institute and the Departments of Medicine and Immunology Duke University School of Medicine Durham NC 27710 USA; ^5^ Duke Human Vaccine Institute Departments of Surgery Molecular Genetics and Microbiology and Immunology Duke University School of Medicine Durham NC 27710 USA; ^6^ Vanderbilt Vaccine Center Vanderbilt University Medical Center Nashville TN 37232 USA; ^7^ Department of Veterinary Biosciences and Diagnostic Imaging College of Veterinary Medicine University of Georgia Athens GA 30602 USA

**Keywords:** antibody therapies, gene therapies, passive immunizations, RNA therapeutics, SARS‐CoV‐2

## Abstract

Despite the success of severe acute respiratory syndrome coronavirus‐2 (SARS‐CoV‐2) vaccines, there remains a clear need for new classes of preventatives for respiratory viral infections due to vaccine hesitancy, lack of sterilizing immunity, and for at‐risk patient populations, including the immunocompromised. While many neutralizing antibodies have been identified, and several approved, to treat COVID‐19, systemic delivery, large doses, and high costs have the potential to limit their widespread use, especially in low‐ and middle‐income countries. To use these antibodies more efficiently, an inhalable formulation is developed that allows for the expression of mRNA‐encoded, membrane‐anchored neutralizing antibodies in the lung to mitigate SARS‐CoV‐2 infections. First, the ability of mRNA‐encoded, membrane‐anchored, anti‐SARS‐CoV‐2 antibodies to prevent infections in vitro is demonstrated. Next, it is demonstrated that nebulizer‐based delivery of these mRNA‐expressed neutralizing antibodies potently abrogates disease in the hamster model. Overall, these results support the use of nebulizer‐based mRNA expression of neutralizing antibodies as a new paradigm for mitigating respiratory virus infections.

## Graphical abstract

Despite the success of vaccination, severe acute respiratory syndrome coronavirus‐2 (SARS‐CoV‐2) remains an ongoing threat worldwide. Monoclonal antibody therapies have proven useful but are expensive and require high systemic doses. This study reports mRNA‐expressed membrane‐anchored antibodies along with an inhalable polymeric nanoparticle formulation that prevent SARS‐CoV‐2 infection in the stringent hamster model.

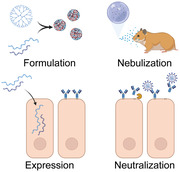

## 1 Introduction

The betacoronavirus severe acute respiratory syndrome coronavirus‐2 (SARS‐CoV‐2) has infected over 270 million individuals to date, and the subsequent disease, COVID‐19, is attributed to more than 5 million deaths globally as of January 2022. Two mRNA‐based vaccines, an adenovirus‐vectored vaccine, and multiple monoclonal antibody (mAb) therapies have been approved or obtained emergency use authorization by the US Food and Drug Administration (FDA) or conditional marketing authorization by the European Union's European Medicines Agency for prevention and treatment, but vaccine hesitancy, lack of sterilizing immunity, and the need for mAb administration within medical settings have underscored the need for self‐administered approaches to limit the spread of the virus. One of the primary drawbacks of passive immunization and treatment using mAbs is the cost, largely due to the high dose of purified, recombinant protein required for efficacy, on the scale of 10–100 mg kg^−1^, and the need for administration within a medical setting.^[^ ^1^, ^2^, ^3^ ^]^ High doses are required, in part, because of the systemic delivery route, as the vast majority of administered antibody never reaches the tissue of interest and remains in circulation.^[^ ^4^ ^]^

Expression of neutralizing mAbs through mRNA is attractive for several reasons. First, unlike DNA and DNA‐based viral vectors, mRNA does not typically enter the nucleus. This reduces the risk of integration with the host genome, as has been reported with adeno‐associated virus vectors.^[^ ^5^ ^]^ Moreover, because mRNA is degraded relatively quickly, long term effects can be minimized.^[^ ^6^ ^]^ Second, a much lower dose of mRNA can be used (0.1–1 mg kg^−1^) when delivered directly to the site of need, the respiratory tract, compared with intravenous or intramuscular mAb dosages. We recently demonstrated that mRNA‐encoded mAbs can be delivered directly to the lungs, producing the mAb in the primary organ target of respiratory pathogens.^[^ ^7^ ^]^ Furthermore, by delivering mRNA rather than the recombinant protein, we were able to encode a glycosylphosphatidylinositol (GPI) membrane anchor into the immunoglobulin G (IgG) heavy chain sequence, allowing for the retention of the antibody in the lung for several weeks.^[^ ^7^, ^8^ ^]^ Our initial demonstration, though, required intratracheal administration, which is not ideal. Here, we demonstrate that nebulization of a polymer‐formulated mRNA‐encoded neutralizing mAb delivered as prophylaxis, abrogated a SARS‐CoV‐2 infection in the hamster model, significantly reducing viral titer, viral RNA levels, lung pathology, and infection‐induced weight loss.

## 2 Results

### 2.1 mRNA Expressed, GPI‐Linked Antibodies Are Anchored to the Plasma Membrane and Potently Inhibit SARS‐CoV‐2

**Figure 1:**
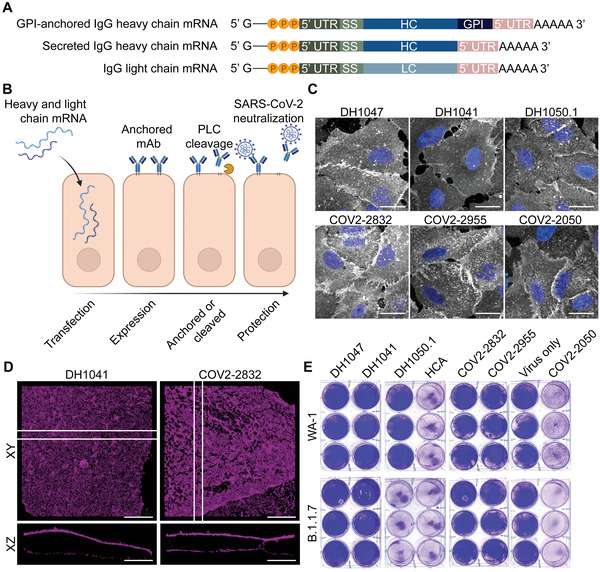
mRNA expressed, GPI‐linked antibodies are anchored to the plasma membrane and potently inhibit SARS‐CoV‐2. A) mRNA schematics encoding GPI‐anchored and secreted IgG heavy chains and the IgG light chain sequences. B) Heavy chain mRNA, with a C‐terminal GPI‐anchor signal, and light chain mRNA is transfected into cells. Anchored IgGs are then displayed on the plasma membrane, can be cleaved by PLC, and then neutralize incoming virus. C) Cells were transfected with mRNA‐expressed anti‐SARS‐CoV‐2 Abs (white) and immunostained at 24 h. Scale bar represents 20 µm. Representative images from *n* = 2 wells per condition. D) Super‐resolution microscopy of mRNA‐expressed anti‐SARS‐CoV‐2 Abs (magenta) 24 h after transfection. Cross sections are represented as the 1 µm region between the white bars. Scale bar represents 7 µm. E) Cells were transfected with mRNA‐expressed Abs 24 h prior to WA‐1 or B.1.1.7 SARS‐CoV‐2 inoculation. HCA mRNA was used as an IgG control. 72 h after infection, cells were analyzed by crystal violet stain for CPE. *n* = 3 wells per condition.

Recently, neutralizing mAbs were discovered using B‐cells from SARS‐CoV‐2 positive individuals.^[^ ^9^, ^10^ ^]^ In our study, three of these antibodies, COV2‐2050, COV2‐2832, and COV2‐2955, from the Crowe laboratory, and DH1041, DH1047, and DH1050.1, from the Haynes laboratory were selected, and in vitro‐transcribed mRNA was produced. To increase half‐life of the expressed proteins, we encoded both an LS mutation (M428L/N434S) and GPI anchor into the heavy‐chain domain encoded by the mRNAs (**Figure** **1**A).^[^ ^7^, ^8^, ^11^ ^]^ The premise is that upon delivery and transfection of the cells in the lung, the mRNA is translated into the IgG protein for the mAb, which is then displayed on the extracellular surface of the plasma membrane (Figure 1B). From there, the mAb remains anchored to the membrane, or the GPI anchor is cleaved by endogenous phospholipase C or D, releasing the mAb into the lung mucosa.^[^ ^12^, ^13^ ^]^ In either location, the antibody could bind and neutralize SARS‐CoV‐2 virions, mitigating infection.

We first verified the expression and membrane localization of the mRNA‐expressed antibodies using both confocal and super‐resolution direct stochastic optical reconstruction microscopy (dSTORM) in A549 cell culture monolayers (Figure 1C,D). As expected, the majority of mAb signal was found on the surface of the plasma membrane in the confocal microscopic images. When examined using dSTORM, the localization was confirmed, and both DH1041 and COV2‐2832 were easily visible above and below the nuclei of transfected cells and well distributed in the membrane. dSTORM was used to confirm the localization as the z‐resolution is greatly enhanced using this technique (≈50 nm) compared with diffraction limited imaging. Interestingly, COV2‐2050 formed long filamentous structures on the surface, likely due to antibody aggregation and thus was not used beyond in vitro testing (Figure S1, Supporting Information). Next, we confirmed that the mRNA‐expressed antibodies retained their neutralizing capacity in a cytopathic effect (CPE) assay using Vero E6 cell monolayer cultures (Figure 1E), and SARS‐CoV‐2 WA‐1 or B.1.1.7 variants. Critically, all the tested mRNA‐expressed SARS‐CoV‐2 neutralizing mAbs inhibited CPE using the WA‐1 wild‐type virus, while DH1050.1 lost efficacy against B.1.1.7 and was thus not investigated in further assays. Moreover, the GPI‐anchored IgG control antibody (human contraceptive antibody, HCA)^[^ ^14^ ^]^ did not protect the cells from infection, demonstrating specificity of the mRNA‐expressed antibodies.

**Figure 2:**
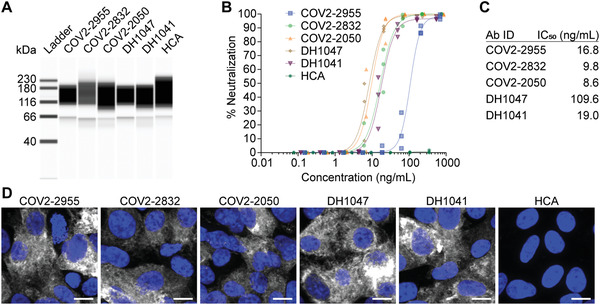
Cleaved mRNA‐expressed antibodies bind and neutralize SARS‐CoV‐2 WA‐1. A) Capillary‐based western blot of PLC‐cleaved mRNA‐expressed Abs. B) SARS‐CoV‐2 WA‐1 neutralization via PLC‐cleaved mRNA‐expressed Abs from (A) (*n* = 3 wells per dilution). C) Neutralizing IC_50_ values of Abs from (A). D) Cells transfected with SARS‐CoV‐2 S mRNA were immunostained at 24 h with PLC‐cleaved Abs (white) from (A). Scale bar represents 10 µm. Representative images from *n* = 2 wells per condition.

Next, the mRNA‐expressed antibodies were cleaved from the cell surface via phospholipase C (PLC) and concentrated. The mRNA‐expressed, PLC‐cleaved antibodies were assessed using capillary‐based western blotting, demonstrating the expected peak at ≈150 kDa (**Figure** **2**A). Subsequently, these same Abs were evaluated in a virus neutralization assay (Figure 2B). In general, all the tested mAbs displayed low IC_50_ values, with a 9.8 or 19.0 ng mL^−1^ IC_50_ value for COV2‐2832 or DH1041, the selected candidates from the Crowe or Haynes laboratories, respectively (Figure 2C). Critically, the IC_50_ values from the RNA‐expressed mAbs were consistent with those of the recombinant IgG protein versions, corroborating our previous reports.^[^ ^8^, ^9^, ^10^ ^]^ Last, these antibodies were used to fluorescently immunostain cells expressing the mRNA‐encoded WA‐1 spike (S) trimer (Figure 2D). All the expressed mAbs immunostained cells expressed the S protein except for the HCA control mAb. Together, these data indicate that the mRNA‐expressed GPI‐linked neutralizing mAbs are anchored to the plasma membrane of transfected cells, specifically bind the S protein, and retain neutralizing capabilities.

### 2.2 Inhaled mRNA‐Expressed Anti‐SARS‐CoV‐2 Antibodies Persist and Are Well Tolerated in Hamster Lungs

After validating the therapeutic cargo in vitro, we next aimed to optimize the expression of mRNAs, delivered via nebulizer, in the golden Syrian hamster, a robust model of SARS‐CoV‐2 infection.^[^ ^15^, ^16^ ^]^ We designed mRNA encoding an IgG for the HCA control antibody with a nanoluciferase (NLuc) fused to the light chain.^[^ ^8^ ^]^ We formulated this mRNA with a poly‐beta amino thio ester (PBATE), a modified and improved polymer from the previously reported poly‐beta amino ester (PBAE) that demonstrated delivery in both mice and hamsters.^[^ ^17^, ^18^ ^]^ We then delivered the polyplexes using an FDA‐approved nebulizer and custom dosing apparatus.

First, we confirmed that the encoded GPI‐anchor was functional and retained the expressed antibody in the lung by measuring the antibody concentration in both the lung and in serum. Hamsters were transfected with 31.3 µg of mRNA encoding, either anchored or secreted (lacking an encoded GPI‐anchor) HCA. At 24 h, we collected lungs and serum and assessed the concentration of HCA (Figure S2, Supporting Information). We observed that while both anchored HCA (aHCA) and secreted HCA (sHCA) concentrations were significantly higher than the control in the lung (340.3 and 274.5 ng g^−1^, respectively), only transfection with the secreted construct resulted in significantly increased serum antibody concentrations (38.9 ng mL^−1^ for sHCA compared to 1.4 ng mL^−1^ for aHCA). This demonstrates that both anchored and secreted antibodies are produced in the lung, as expected following nebulizer‐based mRNA delivery, but only the secreted version is detectable in significant quantities in the blood, indicating that the GPI anchor is retaining the expressed antibody in the lung tissue.

**Figure 3:**
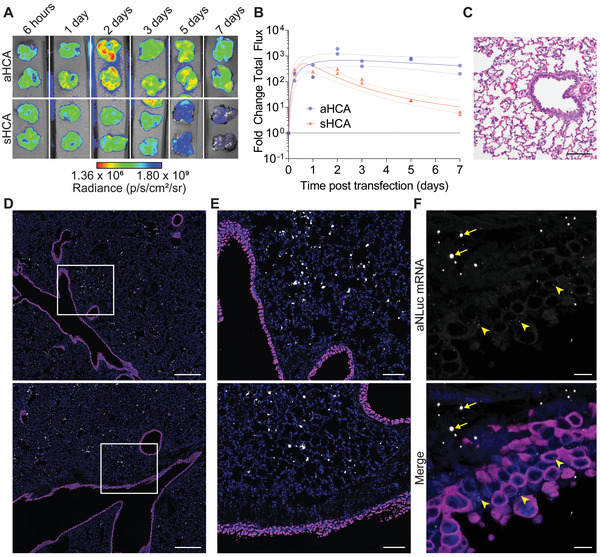
Inhaled mRNA‐expressed anti‐SARS‐CoV‐2 antibodies persist and are well tolerated in hamster lungs. A) Hamsters were treated with 31 µg of nebulized mRNA encoding for either aHCA‐NLuc or secreted HCA (sHCA)‐NLuc. Lungs were excised at indicated time points and frozen at −80 °C until analysis. *n* = 2 hamsters per time point per group. B) Quantification of luminescence in lungs from (A). Line and error bars represent log‐normal regression fit with 95% confidence interval to log‐transformed data. C) Hamster lungs were excised and assessed for pathology via H&E staining at 24 h post‐transfection of 31 µg of aNLuc mRNA. Scale bar represents 100 µm. Representative images from *n* = 2 hamsters per condition. D) Hamster lungs were excised at 4 h post‐transfection of 31 µg of nebulized aNLuc mRNA and fixed and paraffin embedded. Nonciliated bronchiolar cells (magenta) and transfected mRNA (white) were localized using ISH for CC10 or aNLuc, respectively. Scale bar represents 400 µm. Representative images from *n* = 2 hamsters per condition. E) Expanded ISH microscopy images from the white boxes in (D). Scale bar represents 100 µm. F) Spinning disk confocal images of ISH from (D) and (E). Yellow arrowheads indicate small aNLuc mRNA granules in airway cells while arrows indicate larger mRNA granules that would be visible in widefield microscopy. Scale bar represents 10 µm.

Next, we determined the expression kinetics of the anchored antibody mRNA construct with the GPI anchor compared to the secreted version. Using a 31.3 µg dose, we observed peak expression of both the secreted and anchored mAb at 24 to 48 h (**Figure** **3**A,B). As expected, the GPI anchor induced retention of the antibody in the lung, increasing the half‐life from 1.3 to 7.1 days, potentially allowing for delivery of only 1 dose during a SARS‐CoV‐2 infection. This approach would not be onerous compared with other nebulizer‐based treatments, which typically consist of multiple doses a day or a single dose for up to 22 h.^[^ ^19^ ^]^ Finally, we also demonstrated that delivery of PBATE‐formulated mRNA resulted in no observable pulmonary damage or inflammation, as determined by histopathologic evaluation (Figure 3C).

We then assessed the distribution of the nebulized PBATE mRNA formulations in the lungs of hamsters. After delivery of an anchored NLuc reporter mRNA‐encoded antibody, we isolated the lungs and identified nonciliated bronchiolar epithelial cells, in addition to the delivered mRNA, using RNA in situ hybridization (ISH) for Club cell 10 kDa protein (CC10 or Scgb1a1) on whole lung sections using RNAscope (Figure 3D,E). Fluorescent microscopy stitches of whole hamster lungs revealed robust, largely homogenous, delivery of mRNA across all lobes (Figure S3, Supporting Information). Quantification of the mRNA signal revealed that the majority of nebulized, PBATE‐delivered mRNA is observed in the alveolar space, with only 0.81% of the mRNA signal found in the airways and airway epithelial cells. Transfection of the alveolar cells likely could prevent lower respiratory tract infections and severe outcomes, since by day 5 post infection, SARS‐CoV‐2 is largely within the alveolar space and is mostly cleared from the bronchioles in the hamster model.^[^ ^20^, ^21^ ^]^ Finally, we assessed the distribution of nebulized mRNA delivery at higher resolution using spinning disk confocal microscopy. While we observed the same large granules of RNA in the alveolar space as visualized at lower resolution, the increased resolution and contrast revealed small punctate mRNA granules throughout the airway epithelia (Figure 3F). These data, when taken together, indicate that a low dose of mRNA can achieve high expression of durable mAb constructs across much of the hamster lung alveolar and airway epithelial compartments, with minimal pulmonary toxicity.

**Figure 4:**
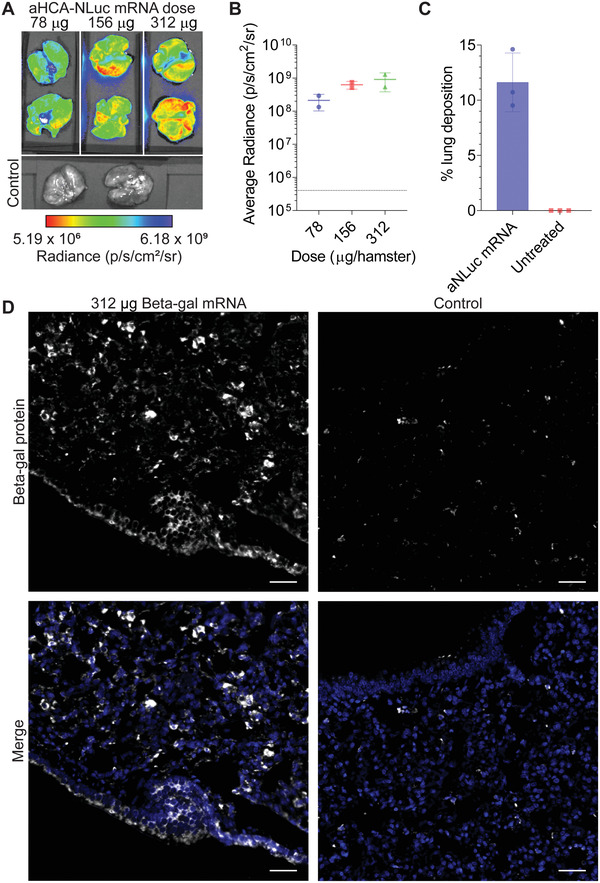
Protein expression characterization of nebulizer‐based pulmonary delivery of concentrated nanoparticles. A) Hamsters were treated with nebulized mRNA encoding for anchored HCA (aHCA)‐NLuc at the indicated doses. Lungs were excised and assessed at 24 h for luminescence. *n* = 2 hamsters per group. B) Quantification of luminescence in lungs from (A). Bars represent mean ± SD. Control lung luminescence indicated by dotted line. C) Hamsters were treated via nebulizer with 312 µg of concentrated aNLuc mRNA. Lungs were assessed immediately by PCR for aNLuc mRNA content. *n* = 3 hamsters per group. Bars represent mean ± SD. D) Hamsters were treated via nebulizer with 312 µg of concentrated mRNA encoding Beta‐gal. Control animals were left untreated. Lungs were excised at 24 h and immunostained for Beta‐gal protein expression (white) and nuclei (blue). Scale bar represents 40 µm. Representative images from *n* = 3 hamsters per condition.

Due to the low minute volume of small rodents, only ≈5.5% of the nebulized dose is actually delivered to the animals’ lungs.^[^ ^22^ ^]^ We hypothesized that concentrating the polyplex formulation would increase the effective dose delivered to the hamster lungs. To determine an appropriate dose of concentrated particles, we delivered 78, 156, or 312 µg of concentrated HCA‐NLuc mRNA particles per hamster. When we measured the luminescence of the lungs at 1 day post‐transfection, we observed an increasing dose response (**Figure** **4**A,B), supporting the delivery of 312 µg of mRNA per animal.

Next, we assessed the amount of mRNA deposited in the lung following nebulization of concentrated particles. To this end, we delivered 312 µg of aNLuc mRNA to hamsters via nebulizer, immediately extracted their lungs, and assessed them for aNLuc mRNA content using PCR. On average, we observed an 11.62% deposition fraction, higher than previously reported (Figure 4C). Based on this deposition, a delivery of 312 µg by nebulizer results in 36.2 µg of mRNA delivered to the lung, or about 0.362 mg kg^−1^ in a hamster. Critically, nebulized delivery to larger animals, such as swine and humans, is closer to 30–50%,^[^ ^23^, ^24^ ^]^ which would drastically reduce the amount of particles required to reach a similar dosage.

Finally, to visualize the distribution of mRNA‐expressed protein in the lung, we delivered 312 µg of beta‐galactosidase (Beta‐gal) encoding mRNA. At 24 h post‐transfection, we then analyzed the lungs with microscopy for Beta‐gal protein expression (Figure 4D). We observed strong intracellular Beta‐gal expression both in the alveolar space and in the airway epithelium, consistent with the mRNA localization we observed above (Figure 3D–F). Overall, these results demonstrate the potent nebulizer‐based delivery and expression of concentrated mRNA polyplexes to the lungs of hamsters.

### 2.3 Nebulized mRNA Expressed GPI‐Anchored Anti‐SARS‐CoV‐2 Antibodies Prevent Infection in Hamsters

**Figure 5:**
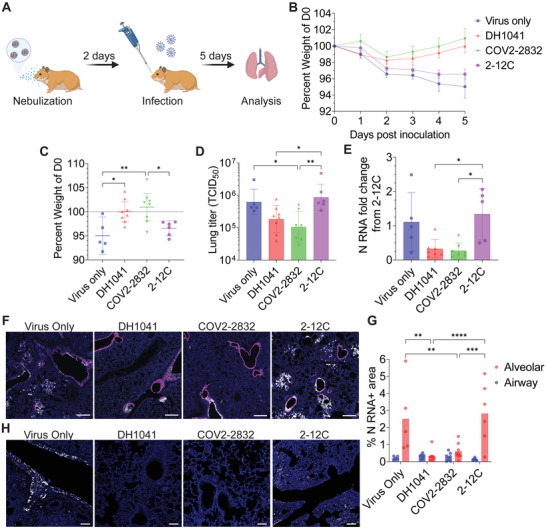
Nebulized mRNA expressed GPI‐anchored anti‐SARS‐CoV‐2 antibodies prevent infection in hamsters. A) Hamsters were dosed with 312 µg of nebulized mRNA encoding, either membrane anchored DH1041 (*n* = 8) or COV2‐2832 (*n* = 7) anti‐SARS‐CoV‐2 antibodies or anchored 2‐12C (*n* = 6) anti‐influenza Ab as an IgG control. One group was left untreated (*n* = 5). 2 days later, all animals were inoculated intranasally with 10^3^ PFU of SARS‐CoV‐2 WA‐1. Weights were monitored over 5 days, and lungs were excised for analysis at day 5. Lungs were split such that the right lung was used for RT‐PCR and plaque assay while the left lung was fixed, paraffin embedded, and sectioned. B) Mean hamster weights over time normalized to the day of infection. Bars indicate mean ± SEM. C–E) Individual hamster weights (C), lung titer (D), and lung normalized viral N RNA (E) on day 5 post infection. Bars indicate mean ± 95% CI for part (C) and mean ± SD for parts (D) and (E). *p < 0.05, **p < 0.005 by one‐way ANOVA with Tukey's multiple comparisons. A subset of animals was selected for N RNA PCR analysis from the DH1041 (*n* = 7) and 2‐12C (*n* = 5) groups. F) Hamster lungs at day 5 were analyzed by ISH for club cell (magenta) and SARS‐CoV‐2 N (white) RNA localization. Scale bar represents 400 µm. G) Quantification of individual percent SARS‐CoV‐2 N RNA positive area within indicated lung cell types from images in (F). Bars indicate mean. **p < 0.005, ***p < 0.0005, ****p < 0.0001 by two‐way ANOVA with Šídák's multiple comparisons. H) Hamster lungs at day 5 were probed with immunostaining for S protein (white). Scale bar represents 100 µm.

Once we established that the mRNA‐expressed mAbs neutralized SARS‐CoV‐2 and optimized the nebulizer‐based delivery of mRNA‐encoded mAbs into hamsters, we then sought to test the prophylactic efficacy of two of the anti‐SARS‐CoV‐2 neutralizing constructs. We delivered mRNA encoding, either COV2‐2832, DH1041, or an IgG control antibody, 2‐12C (an anti‐influenza HA mAb),^[^ ^25^ ^]^ all with a GPI anchor, to hamsters using a nebulizer at a 312 µg dose. Hamsters were then inoculated intranasally 2 days post delivery with 10^3^ PFU of SARS‐CoV‐2 WA‐1 (**Figure** **5**A). Critically, hamsters treated with mRNA‐expressed COV2‐2832 or DH1041 demonstrated dramatic improvement in weight gain from day 2 onwards (Figure 5B). Prophylaxis with COV2‐2832 mRNA had a significant, positive effect on weights at day 5 post‐infection with hamsters gaining an average of 1% body weight compared to an average weight loss of 5% or 3.4% in the virus‐only or 2‐12C mRNA‐treated controls, respectively. Similarly, DH1041 mRNA prophylaxis resulted in no average weight change at day 5 post‐infection, but these weights were significantly higher than those of the virus‐only case (Figure 5C). Lungs from the same hamsters were then analyzed on day 5 post‐infection for viral titer, virus load, and lung histopathology. First, we observed lower viral titers, with an 87% or 81% reduction in TCID_50_ mediated by COV2‐2832 or DH1041, respectively, compared to the 2‐12C control (Figure 5D). We also measured lower viral load in the COV2‐2832‐ or DH1041‐treated hamsters by qPCR for total nucleocapsid (N) RNA, with an 80% or 76% reduction, respectively, compared to the 2‐12C control (Figure 5E). In addition, the lungs from the 2‐12C‐treated, virus‐only, and COV2‐2832‐ or DH1041‐treated animals were evaluated using RNAscope for viral N RNA (Figure S4, Supporting Information). Consistent with our other results, we observed dramatically reduced signal for SARS‐CoV‐2 N RNA in the alveolar space in the both the DH1041‐ and COV2‐2832‐treated animals (Figure 5F). When the percent area of signal was quantified and stratified by spatial localization, we observed an 88% or 79% decrease in signal in the alveolar space with DH1041 or COV2‐2832 treatment compared to the 2‐12C control, respectively, while in the larger airways, there was little effect (Figure 5G). Currently, we believe this finding points to the limitation of the nebulizer‐based approach in rodents, where the deposition of small droplets is clearly directed to the alveolar space. In larger species, this issue should be alleviated and allow for greater targeting of the entire respiratory tract. Finally, we analyzed the distribution of the SARS‐CoV‐2 spike (S) protein in the lungs, finding significantly reduced staining in both the airways and alveolar spaces in the DH1041 or COV2‐2832 treated animals compared to the 2‐12C and virus only control groups (Figure 5H). Overall, these results suggest that prophylaxis with mRNA encoding membrane‐anchored anti‐SARS‐CoV‐2 antibodies significantly reduces hamster weight loss, viral replication, and viral load in the lungs.

### 2.4 Nebulized mRNA Expressed GPI‐Anchored Anti‐SARS‐CoV‐2 Antibodies Prevent Significant Pathology in Hamster Lungs

**Figure 6:**
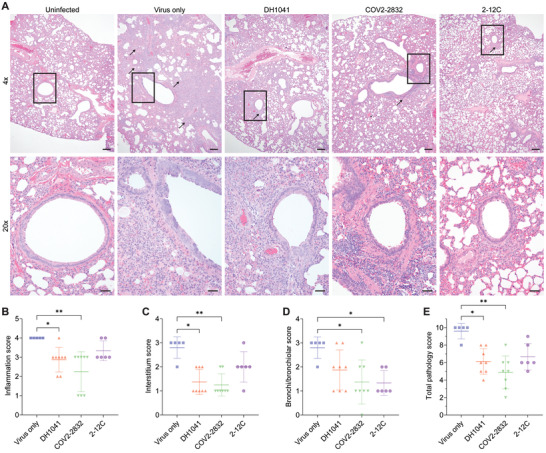
Nebulized mRNA expressed GPI‐anchored anti‐SARS‐CoV‐2 antibodies prevent significant pathology in hamster lungs. Hamster lungs were stained with H&E and scored for pathology. A) Representative images of lung pathology. Scale bar represents 200 µm (top, 4× images) or 50 µm (bottom, 20× images). Arrows indicate alveolar consolidation extending from bronchi and bronchioles. Boxes indicate 20× inset within 4× image. B) Inflammation, C) interstitium, D) bronchi/bronchiolar, and E) total pathology scores from individual hamsters. Bars represent mean ±SD. *p < 0.05, **p < 0.005 by Kruskal–Wallis one‐way ANOVA with multiple comparisons.

We then analyzed the lungs of these hamsters for pathology, finding that both the DH1041 and the COV2‐2832 mRNA‐treated animals showed decreased lung pathology when analyzed for alveolar consolidation, alveolar septal wall thickening, and bronchiolar epithelial hyperplasia (**Figure** **6**A), as indicated by the significantly lower pathology scores (up to 51%) compared to the virus only group (Figure 6B–E). Interestingly, animals treated with COV2‐2832 mRNA also had a 51% lower bronchi/bronchiolar score. This indicates that while mRNA delivery to the airway epithelium is low compared the alveolar space, there is enough antibody present to decrease bronchiolar hyperplasia. Importantly, this result could be impacted by the day 5 assessment, as the virus is mostly cleared from the bronchi at this timepoint.^[^ ^15^ ^]^ Collectively, these results suggest that prophylaxis with mRNA‐expressed COV2‐2832 or DH1041 can considerably reduce disease induced by SARS‐CoV‐2 infection in the hamster model.

### 2.5 Correlation of SARS‐CoV‐2 Infection Metrics in the Hamster Model

**Figure 7:**
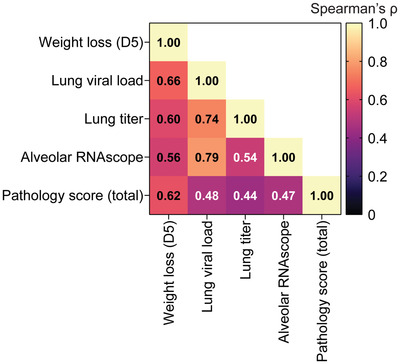
Correlation of SARS‐CoV‐2 infection metrics in the hamster model. Heat map of Spearman's correlation coefficient (*r*) between infection metrics across all treatment groups.

Finally, to better assess the correlates of viral protection in the hamster SARS‐CoV‐2 challenge model, we quantified the multivariate correlation of day 5 weight loss, lung viral load (by PCR), lung viral titer (TCID_50_), percent SARS‐CoV‐2 N RNA‐positive alveolar area, and total pathology score (**Figure** **7**). Overall, we found that percent weight loss at day 5 displayed the most consistent correlation with the other variables, varying only from 0.56 (alveolar RNAscope) to 0.66 (lung viral load) using Spearman correlation. As expected, we observed the highest correlations between lung viral load and both alveolar RNAscope and lung viral titer. Finally, the total pathology score showed the weakest overall correlations to the other metrics, except for weight loss. Overall, these data indicate that 1) weight loss is a good general measure of animal health, and 2) the lung pathology score is likely less dependent on the presence of replicating virus and more dependent on the severity of immune responses in the infected animals.

## 3 Conclusion

Four antibody treatments have obtained EUA since the start of the COVID‐19 pandemic, including the mAbs for therapy casirivimab + imdevimab (EUA withdrawn), bamlanivimab + etesevimab (EUA withdrawn), and sotrovimab and the prophylactic mAbs tixagevimab + cilgavimab. All these antibodies are typically administered in a medical setting via the IM or IV route with dosages of over 7 mg kg^−1^. The use of mRNA to express mAbs has several benefits, including lower dosages (≈0.16 mg kg^−1^ in vivo), potential for lower cost, and the potential for cold‐chain independence, among others. A phase 1 proof‐of‐concept trial of an mRNA‐encoded antibody has been reported, using the variable genes encoding a chikungunya virus‐specific neutralizing mAb.^[^ ^26^ ^]^ That study used IV infusion of lipid nanoparticle (LNP) encapsulated mRNA, which is expected to express protein principally in the liver tissues. Alternatively, mRNA‐encoded mAbs could be delivered directly to the mucosal surface, where the expressed mAbs can neutralize pathogens that target those tissues.^[^ ^7^, ^8^ ^]^ Such local delivery results in concentrated amounts of mAb in the compartment of interest, removing the need for systemic infusion of high doses of IgG protein or LNPs carrying mRNA. Compared to previous reports using IV delivery of neutralizing mAbs in hamsters, inhaled mRNA‐expressed mAbs yield competitive results but with much lower doses, exhibiting robust weight gain, and significant drops in lung titer, viral RNA by qPCR, and percent SARS‐CoV‐2 RNA+ area via RNAscope in the alveolar space.^[^ ^27^, ^28^, ^29^, ^30^ ^]^ It should be noted that in the present work, we used human antibodies in the hamster lung, and therefore these antibodies do not exhibit optimal Fc‐mediated functions mediated through interactions with Fc gamma receptors. Future experiments using species‐matched Fc domains could allow for improved efficacy.

As of this writing, three vaccines have been issued an EUA in the US for prevention of SARS‐CoV‐2 infection, two of which are mRNA‐based, and one is adenovirus‐vectored, with the Pfizer and Moderna vaccines given full approval. Two small molecule drug treatments, molnupiravir and nirmatrelvir, have been assigned EUA for treatment. Both the vaccines and new drugs are vital to addressing the ongoing pandemic. However, vaccines rely on the immune response of the inoculated individual and high similarity of the vaccine antigens to the S protein of actively circulating variants. Passive immunization with neutralizing mAbs, either directly or through mRNA expression, circumvents the need for the host immune system to provide optimal response to achieve immunity. Moreover, both protein and mRNA‐based IgG passive immunization routes confer protection much faster than active immunization, which is critical when the target pathogen is actively circulating. Furthermore, both mAb therapy and current vaccines require a practitioner for dosing. The nebulizer‐based approach presented here has the potential for self‐administration at home, an important benefit that could improve drug distribution and access.

Here, we detail the use of mRNA‐expressed anti‐SARS‐CoV‐2 antibodies to prevent SARS‐CoV‐2 related disease. We demonstrated that mRNA‐expressed mAbs retain the neutralizing capacity of the parental antibodies and that PBATE formulations can deliver sufficient mRNA quantity to the lungs of hamsters to abrogate disease. Overall, both mRNA‐expressed COV2‐2832 and DH1041 represent promising prophylactic options in the ongoing fight against the COVID‐19 pandemic and are a clear complementary prophylactic strategy to the therapies currently in use.

## 4 Experimental Section

### Cell Lines and Viruses

Cell lines were purchased from the American Type Culture Collection. A549 (CCL185) and Vero E6 cells (C1008) were grown in Dulbecco's Modified Eagle Medium (DMEM) supplemented with 5% fetal bovine serum (FBS) and 1% penicillin–streptomycin. Microscopy and PLC release studies were performed in A549 cells. SARS‐CoV‐2 studies were performed in Vero E6 cells.

SARS‐CoV‐2 (USA‐WA1/2020 and hCoV‐19/USA/CA_CDC_5574/2020) was obtained from BEI Resources. Viral stocks were generated by infecting Vero E6 cells at ≈95% confluency in 150 cm^2^ flasks with SARS‐CoV‐2 at an MOI of 0.1 PFU per cell. At 68 h after infection, supernatants were collected, pooled, and centrifuged at 400 × *g* for 10 min. The resulting stock was aliquoted, titered, and stored at −80 °C for further use. All work with live SARS‐CoV‐2 was performed inside a certified Class II Biosafety Cabinet in a Biological Safety Level (BSL)‐3 laboratory in compliance with all state and federal guidelines and with the approval of the University of Georgia Institutional Biosafety Committee.

### In Vitro Transcribed RNA Synthesis

Plasmid templates were designed using Geneious software, and codon optimized and ordered from GenScript. Plasmids were linearized with Not‐I HF (New England Biolabs) overnight at 37 °C. Linearized templates were purified by sodium acetate (Thermo Fisher Scientific) precipitation and rehydrated with nuclease‐free water. In vitro transcription was performed overnight at 37 °C using a HiScribe T7 Kit (NEB) following the manufacturer's instructions (complete N1‐methyl‐pseudouridine modification). The resulting RNA was treated with DNase I (Aldevron) for 30 min and purified using lithium chloride precipitation (Thermo Fisher Scientific). The RNA was heat denatured at 65 °C for 10 min before capping with a Cap‐1 structure using guanylyl transferase and 2′‐O‐methyltransferase (Aldevron). mRNA was then purified by lithium chloride precipitation, treated with alkaline phosphatase (NEB), and purified again. mRNA concentration was measured using a Nanodrop. mRNA stock concentrations were 1–3 mg mL^−1^ and were stored at −80 °C until use. Purified mRNA products were analyzed by gel electrophoresis to ensure purity.

### In Vitro Transfections

Vero E6 or A549 cells were transfected using Lipofectamine Messenger Max (Thermo Fisher), according to the manufacturer's instructions. Cells in 24‐well plates were transfected with 1 µg/well of total mRNA while cells in 6‐well plates were transfected with 5 µg/well of total mRNA. For mRNA encoding whole IgG, heavy chain and light chain mRNAs were combined in a 4:1 mass ratio for equimolar conditions.

### Confocal Microscopy

Cells in #1.5 borosilicate coverslip bottom plates (Cell‐vis) were fixed in 4% paraformaldehyde for 10 min, permeabilized with 0.2% Triton‐X for 5 min, and blocked with 5% BSA for 30 min. For staining cells transfected with SARS‐CoV‐2 S mRNA, the PLC‐cleaved mAbs were used at a 1 µg mL^−1^ final concentration. The secondary antibodies used were Alexa Fluor Plus 647 goat anti‐human (1:250, Thermo Fisher Scientific, A48279). All antibodies were incubated for 30 min at 37 °C. Cells were routinely stained with DAPI before mounting with Prolong Gold (Thermo Fisher Scientific). Images were acquired with a Hamamatsu Flash 4.0 v2 sCMOS camera on a PerkinElmer UltraView spinning disk confocal microscope mounted to a Zeiss Axiovert 200 M body with a 63× NA 1.4 plan‐apochromat objective lens. Images were acquired with Volocity Acquisition Software (version 6.3.3, PerkinElmer) with z‐stack intervals of 200 nm. Images were linearly contrast enhanced for visual clarity. Images were uniformly contrast enhanced across an experiment.

### dSTORM

Images were acquired using a Bruker Vutara 352 with a 63× NA 1.2 plan‐apochromat water immersion objective lens kept at room temperature. Samples were immunostained as above without permeabilization and without nuclear counterstain since the 405 laser was used for dSTORM photoactivation. Samples were imaged in a buffer to enhance fluorophore blinking which contains: 50 mm Tris‐HCl (pH 8.0), 10 mm NaCl, 10% glucose, 20 mm cysteamine, 1% 2‐Mercaptoethanol, 169 AU mL^−1^ glucose‐oxidase, and 1404 AU mL^−1^ catalase. Imaging buffer was prepared fresh daily and replaced after ≈2 h. All images were acquired and analyzed using the Vutara SRX software. All images were processed using a denoise level of 0.2.

### CPE Assay

After an overnight incubation at 37 °C and 5% CO_2_, plates were transferred to the BSL‐3 for infection. The medium was removed, and cells were infected with SARS‐CoV‐2 for 45–60 min at an MOI of ≈0.1 PFU per cell. After infection, cells were overlaid with 1× DMEM with 1% FBS containing 1.2% Avicel RC‐581 and incubated for 72 h. The overlay was removed and cells were rinsed with 1× PBS and fixed/stained with a crystal violet solution containing 2% methanol and 4% formaldehyde for 10 min.

### PLC Release and Analysis of Anchored mAbs

PLC (Sigma) was reconstituted with 1% BSA in PBS (w/Ca^2+^ and Mg^2+^) at a 2.5 units mL^−1^ final concentration. Transfected cells were first washed with PBS before PLC solution was added, and the cells were incubated for 1 h at 37 °C. The solution was collected and centrifuged at 1000 × g for 10 min to remove cell debris. Amicon Ultra‐15 Centrifugal Filters (MWCO 100 kDa, Millipore) were used to concentrate the supernatant at 4000 × *g* for 10 min. The concentrated antibody solutions were assessed with an automated capillary‐based western blot system, Jess (Protein Simple), according to the manufacturer's instructions. The samples were separated from 0 to 440 kDa and reacted with a HRP‐conjugated anti‐human IgG, followed by the treatment of a chemiluminescent substrate. The chemiluminescence was measured, and the normalized intensities were plotted.

### SARS‐CoV‐2 Neutralization Assay

Vero E6 cells were plated on 96‐well flat‐bottom plates at ≈10 000 cells per well and allowed to attach for 24 h at 37 °C/5% CO_2_. On a separate plate, antibody preparations were subjected to half‐log_10_ serial dilutions in DMEM 1% heat‐inactivated FBS in a final volume of 100 µL. After dilutions were made, a single concentration of live SARS‐CoV‐2 in 100 µL were added to each well containing diluted antibodies (≈600 PFU/well for wild‐type virus WA‐1 [MOI = 0.06] and ≈1000 PFU/well of B.1.1.7 variant [MOI = 0.1]). These concentrations of virus were confirmed empirically as the minimum amounts that result in >95% killing of cells after 72 h. The mixture of virus and antibodies was then incubated for 1 h at 37 °C/5% CO_2_. All medium was removed from the plate of cells, and the entire 200 µL of virus and antibodies were transferred onto the cells. The plates were then placed at 37 °C/5% CO_2_ for 72 h. Following this incubation, the medium was removed, and any remaining/live cells were stained with a crystal violet/fixative solution for 10 min and photographed. Uninfected cells and virus‐infected (no antibodies) controls were included on every plate. Neutralization was measured using % crystal violet area positive per individual well in Qupath (version 0.3.0). IC_50_ values were quantified using 4‐PL regression in Prism (Graphpad, Prism version 9.3.1).

### Polymer Synthesis

mRNA was formulated for nebulizer‐based delivery using a hyperbranched PBAE as previously described with minor modifications.^[^ ^17^, ^18^ ^]^ Diacrylate and amine monomers were purchased from Sigma‐Aldrich. To synthesize hyperbranched hDD90‐118, acrylate:backbone amine:trifunctional amine monomers were reacted modifying the ratio at 1:0.59:0.40. Monomers were stirred in anhydrous dimethylformamide at a concentration of 150 mg mL^−1^ at 40 °C for 4 h and then 90 °C for 48 h. The mixtures were allowed to cool to 30 °C, and end cap amine was added at 1.0 molar equivalent relative to the acrylate and stirred for an additional 24 h. The polymers were purified by dropwise precipitation into cold anhydrous diethyl ether spiked with glacial acetic acid, vortexed, and centrifuged at 1250 × *g* for 2 min. The supernatant was discarded, and the polymer was washed twice more in fresh diethyl ether and dried under vacuum for 48 h. This step was repeated until the supernatant in the precipitation process became transparent. Polymers were stored at −20 °C until use.

### Animal Studies and Safety

Hamster delivery optimization experiments were performed at the Georgia Institute of Technology. 4‐week‐old male LVG Golden Syrian Hamsters (Charles River Laboratories) were maintained under pathogen‐free conditions in individually ventilated and watered cages kept at negative pressure. Hamsters were kept in rooms on a 12 h light/dark cycle with ambient temperature between 21.1 and 22.8 °C with 35–50% relative humidity. Food was provided to hamsters ad libitum. Animals were acclimatized for at least 6 days before beginning experiments. Animals were randomly distributed among experimental groups. Animals were sacrificed by CO_2_ asphyxiation.

All animals were cared for according to the Georgia Institute of Technology Physiological Research Laboratory policies and under ethical guidance from the university's Institutional Animal Care and Use Committee following National Institutes of Health (NIH) guidelines.

Hamster infections were performed at the University of Georgia. Outbred male LVG Golden Syrian hamsters, 3–4 weeks of age, were obtained from Charles River Laboratories. Hamsters were housed inside an animal BSL‐3 room in a HEPA‐filtered cage/rack system and provided food and water ad libitum. Animals were randomly assigned to groups by animal care staff blinded as to study design and treatment. Hamsters were acclimatized before use. Animals were cared for according to the University of Georgia Animal Health Research Center policies and under ethical guidance from the university's Institutional Animal Care and Use Committee following NIH guidelines (A100169).

### In Vivo Transfections

Before delivery to animals, 100 mm of sodium acetate pH 5.0 was used to both solubilize the hyperbranched PBAE at 50 mg mL^−1^ and dilute mRNA to 1 mg mL^−1^ before mixing. The final concentration of the mRNA was 0.5 mg mL^−1^, and the PBAE was used at a 50× molar ratio to the mRNA. The tubes were vortexed briefly to mix and incubated at room temperature for 10 min.

For experiments involving concentrated formulations, the particles were filtered using a 30 kDa cut‐off centrifugal filter (Amicon Ultra) for 30 min at 4 °C at 2000 × *g*. Filters were then inverted into a new tube, and retentate was collected by centrifugation for 5 min at 4 °C at 1000 × *g*. The volume of the retentate was then measured and used to calculate the volume of delivery needed to achieve the indicated doses.

Hamsters were loaded into a custom‐built nose‐only exposure system constructed of a clear PVC tee and animal restraints (CODA Large Mouse Holder, Kent Scientific). These were connected using a custom 3D‐printed nose cone (3D Printing Tech) made of a flexible thermoplastic urethane material. The nebulizer (Aerogen Aeroneb Solo, Tri‐anim) was then placed on the upward facing port of the tee. To account for the tidal volume in hamsters, doses were added dropwise to the nebulizer at a rate of 62.5 µL per hamster per droplet.

### Luminescence Imaging

After euthanasia, whole lungs were collected and rinsed with PBS. For time course studies, lungs were frozen on dry ice and kept at −80 °C until all time points were complete. Lungs were then placed into a solution of Nano‐Glo substrate solution (Promega) diluted 50‐fold in PBS. Lungs were incubated for 5 min and then placed onto black paper and imaged with an IVIS Spectrum CT (PerkinElmer). Lung luminescence was then quantified using Living Image software (version 4.7.4, PerkinElmer).

### Deposition Quantification by PCR

After euthanasia, lungs were collected into Trizol (Invitrogen) in C tubes (Miltenyi) and kept frozen at −80 °C until analysis. Lungs were thawed and processed on a Miltenyi GentleMACS using the RNA_01 program. Chloroform phase separation was performed, and the aqueous fraction was combined with 70% ethanol. RNA was then extracted using conventional centrifugal columns (RNeasy Plus Mini Kit, Qiagen). Complementary DNA was generated according to manufacturer's instructions using the RT^2^ first‐strand kit (Qiagen) using 1 µg of total RNA. qPCR experiments were performed using the FastAdvanced Master Mix (Thermo Fisher Scientific). Copy number was determined using an aNLuc DNA oligo standard (IDT). Experiments were performed using a QuantStudio7 Flex thermal cycler (Applied Biosystems). The percent deposition was then calculated using the following formula:

(1) $$\frac{n\times V_{\mathrm{cDNA}}}{V_{\mathrm{PCR}}}\times \frac{V_{\mathrm{Trizol}}}{V_{\mathrm{extracted}}}\times \frac{{\mathrm{MW}}_{\mathrm{aNLuc}\;\mathrm{mRNA}}}{N_{A}}$$

where *n* was the copy number in a single reaction, *V*_cDNA_ was the total volume of cDNA, *V*_PCR_ was the volume of cDNA used in a single reaction, *V*_Trizol_ was the total volume of Trizol that the lungs were homogenized in, *V*_extracted_ was the volume of lung homogenate used in the RNA extraction, MW_aNLuc mRNA_ was the MW of aNLuc mRNA, and *N*_A_ was Avogadro's number.

### HCA ELISA

Lungs were collected into 1× PBS in C tubes (Miltenyi) and processed by a Miltenyi GentleMACS Dissociator with the RNA_01 program. The homogenized tissue samples were transferred into 15 mL conical tubes and centrifuged for 20 min at 2000 × g to remove the supernatant. The pellets were resuspended with 2.5 U mL^−1^ of PLC (Sigma‐Aldrich, P5542) in 1× PBS, followed by 1.5‐h incubation at 37 °C with a tube rotisserie. After incubation, the samples were centrifuged for 20 min at 2000 × g. The supernatants were collected and diluted with a sample diluent provided in IgG Human SimpleStep ELISA kit (Abcam). ELISA was then conducted based on the manufacturer's protocol. Serum samples were also diluted with the sample diluent, and human IgG concentrations in the serum were measured using the same ELISA.

### Histology and ISH

Lungs were removed and incubated in 4% paraformaldehyde overnight at 4 °C. Paraffin embedded 5 µm sections were prepared by HistoWiz. Hematoxylin and eosin (H&E) staining, and imaging, were performed by HistoWiz.

Delivered mRNA and endogenous mRNA were visualized using RNAscope Multiplex Fluorescent Reagent Kit v2 (Advanced Cell Diagnostics 323136) according to manufacturer's instructions. A custom probe set was designed against the synthetic aNLuc mRNA sequence (ACD 879571). To distinguish hamster lung airways, probes for secretoglobin (Scgb1a1, ACD 1058131‐C2) were used. To determine SARS‐CoV‐2 RNA localization, probes for N RNA (ACD 863831) were used. Lung tissue was scanned by the Emory Winship Cancer Tissue and Pathology core on a Perkin Elmer Vectra Polaris slide scanner. Images were processed and quantified using QuPath 0.3.0 software, and figure images were produced using QuPath. For whole lung figures, an ROI was drawn around the lung in Adobe Illustrator to mask the region of the slide surrounding the tissue that contained the fluorescent marker signal used by the Vectra Polaris to generate the region to tile scan.

To quantify RNA‐positive regions in mouse lungs using QuPath, an ROI was drawn by hand around the lung regions, excluding the trachea, heart, and other ancillary tissues. An ROI was then drawn by hand around the airways and airway cells in each section, delineated by the ISH airway marker staining. Percent RNA‐positive area was then found by QuPath within each region and plotted.

For high‐resolution confocal imaging of RNA ISH in the hamster lungs, slides were imaged using an Andor BC43 spinning disk confocal microscope with a 60× Plan‐Apochromat NA1.40 objective lens. Images were analyzed using Imaris 9.7.2 (Bitplane).

### Beta‐Gal Protein Staining

Beta‐gal protein was stained using the Tyramide SuperBoost kit with minor modifications (Thermo Fisher). Briefly, lungs were collected in 10% formalin and paraffin‐embedding and 5 µm section preparation was performed by the Cancer Tissue Pathology core at Emory University. Slides were then baked at 60 °C for 1 h followed by two xylene and ethanol washes each. Slides were then dried for 10 min at 60 °C and incubated with hydrogen peroxide for 1 h to remove endogenous peroxidase activity. Antigen retrieval was performed by boiling the slides in a low pH antigen retrieval solution (Thermo Fisher) for 15 min. Slides were then washed in water and 100% ethanol. Slides were dried again, and a barrier was drawn (Vector Labs, H‐4000) and allowed to dry for 5 min. Samples were then washed three times in PBS, blocked for 1 h using 5% BSA and 10% goat serum, and incubated overnight at 4 °C with 8 µg mL^−1^ of a rabbit anti‐Beta‐gal antibody (Thermo Fisher, A‐11132). Slides were washed three times with PBS and incubated with the SuperBoost anti‐rabbit poly‐HRP secondary for 1 h before another three washes. Slides were incubated with SuperBoost reaction buffer using OPAL 690 for 10 min followed by reaction stop buffer for 10 min. Slides were washed three times, and nuclei were stained using Spectral DAPI (Akoya Biosciences). Slides were then mounted with Prolong Gold (Thermo Fisher). Slides were scanned and analyzed using the same system and software as above.

### Hamster SARS‐CoV‐2 Infections

Hamsters were anesthetized by intraperitoneal injection of a mixture of 100 mg kg^−1^ of ketamine/5 mg kg^−1^ of xylazine. After loss of toe pinch reflex, SARS‐CoV‐2 was administered to each hamster via intranasal route in a total volume of 50 µL. Animals were then administered reversal agent (atipamezole, 0.15 mg kg^−1^) and placed on a heating pad until able to right themselves. Body weights and clinical signs were checked and recorded daily. For sample collection, hamsters were anesthetized as described above and administered pentobarbital (100 mg kg^−1^) via intraperitoneal injection. After exsanguination and pneumothorax, tissues were collected aseptically for analyses.

Whole lungs from individual hamsters were placed in 2 mL of DMEM 1% FBS containing antibiotics/antimycotics (D1) in C tubes and homogenized using a GentleMACS machine at “lung 2” setting (Miltenyi). After centrifugation for 10 min at 1000 × *g*, supernatant was removed, and remaining homogenates were resuspended in Trizol for RNA extraction. Chloroform‐based phase separation and RNA precipitation were then performed followed by two ethanol washes.

### SARS‐CoV‐2 Lung Titer

After euthanasia, whole lungs were collected and homogenized in 2‐mL of DMEM 1% heat‐inactivated FBS using a Miltenyi gentleMACS Dissociator. Cell debris were centrifuged at 1000 × *g* for 10 min and the cleared lysates were collected and stored at −80 °C. To determine viral titers, Vero E6 cells were plated on 96‐well flat‐bottom plates at ≈10 000 cells per well and allowed to attach for 24 h at 37 °C/5% CO_2_. On a separate plate, cleared lysates were subjected to serial dilutions in DMEM 1% heat‐inactivated FBS in a final volume of 200 µL. All medium was removed from the plate of cells, and the entire 200 µL of diluted lysates were transferred onto the cells. The plates were then placed at 37 °C/5% CO_2_ for 72 h. Following this incubation, the medium was removed and any remaining/live cells were stained with a crystal violet/fixative solution for 10 min and photographed. Uninfected cells and virus‐infected (no antibodies) controls were included on every plate. TCID50 was measured using % crystal violet area positive per individual well in Qupath (version 0.3.0). TCID_50_ values were quantified using 4‐PL regression in Prism (Graphpad, version 9.3.1).

### SARS‐CoV‐2 Lung Viral Load

After total RNA quantification by Nanodrop, cDNA was prepared using the High‐Capacity cDNA reverse transcription kit (The Applied Biosystems, Thermo Fisher Scientific). qPCR experiments were performed using the FastAdvanced Master Mix (Thermo Fisher Scientific). The fold change of the SARS‐CoV‐2 N gene was calculated using the CDC‐approved N1 primer/probe set (2019‐nCoV_N1) and a 18S primer/probe as endogenous control.^[^ ^31^ ^]^ Experiments were performed using a QuantStudio7 Flex thermal cycler (Applied Biosystems).

### SARS‐CoV‐2 Lung Pathology Scoring

Tissue section slides were deparaffined and stained with H&E. Lung sections were scored blindly based on the following scoring parameters by an ACVP board certified veterinary pathologist. Images were taken on an Olympus BX43 light microscope with a DP72 digital camera and CellSens standard software at 4× and 20×. The total possible score was 10. Histologic changes were characterized by alveolar consolidation that extended from bronchi and bronchioles (inflammation score), consistent with a bronchointerstitial pneumonia. Consolidated areas had collapse of alveoli with infiltration of lymphocytes and macrophages. Type II cell hyperplasia was prominent in more severely affected lungs. Extending from areas of consolidation, alveolar septal walls were thickened due to mononuclear cell infiltration and type II pneumocyte hyperplasia (interstitium score). The inflammation and interstitium changes were scored based on distribution and percent of lung affected based on the following: focal in one lobe (1), focal in more than one lobe (2), patchy distribution affecting <25% total lung area (3), patchy to generalized distribution affecting 25–75% lung area (4). Varying degrees of bronchiolar epithelial hyperplasia (bronchi/bronchiole score) were noted and scored as mild (1), moderate (2), or severe (3).

### Statistics

All experiments were represented as a mean of independent replicates as indicated. Power analyses for group sizes were calculated using G*Power (version 3.1, University of Dusseldorf). Data were analyzed and plotted using Prism software (GraphPad, version 9.3.1). Statistical analyses were performed between groups using either ordinary one‐way or two‐way analysis of variance (ANOVA) as specified in individual figure captions with the indicated multiple comparisons test.

## Conflict of Interest

D.V., C.Z., and P.J.S. are cofounders of Tether Therapeutics Inc. These arrangements are managed by Emory University. J.E.C.J. has served as a consultant for Luna Innovations, Merck, and GlaxoSmithKline, is a member of the Scientific Advisory Board of Meissa Vaccines, and is founder of IDBiologics. The Crowe lab has received unrelated sponsored research agreements from Takeda Pharmaceuticals, IDBiologics, and AstraZeneca. These arrangements are managed by Vanderbilt University Medical Center.

## Supporting information

Supporting Information

## Data Availability

The data that support the findings of this study are available from the corresponding author upon reasonable request.
