# Supplementary material for: Nebulized mRNA‐Encoded Antibodies Protect Hamsters from SARS‐CoV‐2 Infection
Source: Adv Sci (Weinh). 2022 Oct 31;9(34):2202771. doi: 10.1002/advs.202202771 (PMC9731714; doi:10.1002/advs.202202771)
Supplement: Supplementary file 1 — Supporting Information [file ADVS-9-0-s001.pdf]

ADVANCED SCIENCE
Open Access

Supporting Information

for *Adv. Sci.*, DOI 10.1002/advs.202202771

Nebulized mRNA-Encoded Antibodies Protect Hamsters from SARS-CoV-2 Infection

Daryll Vanover, Chiara Zurla, Hannah E. Peck, Nichole Orr-Burks, Jae Yeon Joo, Jackelyn Murray, Nathan Holladay, Ryan A. Hobbs, Younghun Jung, Lorena C. S. Chaves, Laura Rotolo, Aaron W. Lifland, Alicia K. Olivier, Dapeng Li, Kevin O. Saunders, Gregory D. Sempowski, James E. Crowe Jr., Barton F. Haynes, Eric R. Lafontaine, Robert J. Hogan* and Philip J. Santangelo**

Supporting Information

Nebulized mRNA-encoded antibodies protect hamsters from SARS-CoV-2 infection

Daryll Vanover, Chiara Zurla, Hannah E. Peck, Nichole Orr-Burks, Jae Yeon Joo, Jackelyn Murray, Nathan Holladay, Ryan A. Hobbs, Younghun Jung, Lorena C.S. Chaves, Laura Rotolo, Aaron W. Lifland, Alicia K. Olivier, Dapeng Li, Kevin O. Saunders, Gregory D. Sempowski, James E. Crowe Jr., Barton F. Haynes, Eric R. Lafontaine*, Robert J. Hogan*, Philip J. Santangelo*

Figure S1.

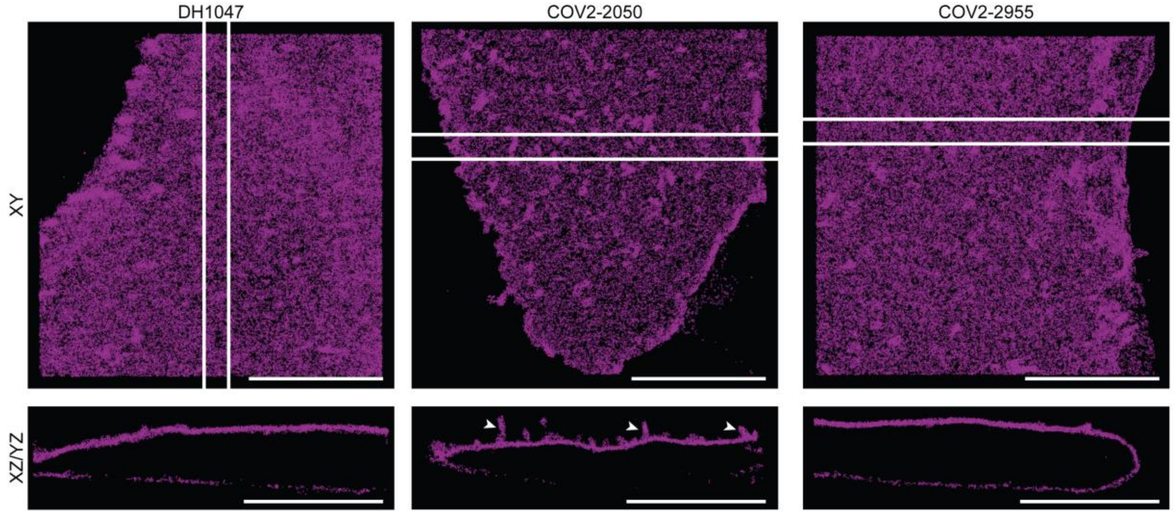

Super-resolution microscopy of mRNA-expressed anti-SARS-CoV-2 Abs (magenta). Cross sections are represented as the 1 µm region between the white bars. Scale bar represents 7 µm. Arrowheads indicate protrusions on the upper plasma membrane of cells transfected with 2050 Ab mRNA.

Figure S2.

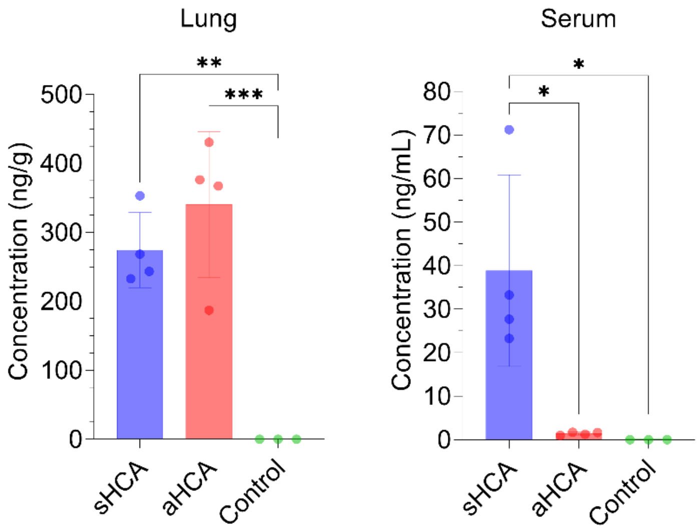

Hamsters were treated with either aHCA or sHCA mRNA via nebulizer. At 24 hours, serum and lungs were collected. All lungs were treated with PLC to cleave membrane-anchored antibodies. Lungs and sera were then assayed by human IgG ELISA for HCA quantification. Bars represent mean ± SD. * indicates p<0.05, ** indicates p<0.01, *** indicates p<0.001. n = 4 hamsters for sHCA and aHCA groups. n = 3 hamsters for control group.

Figure S3.

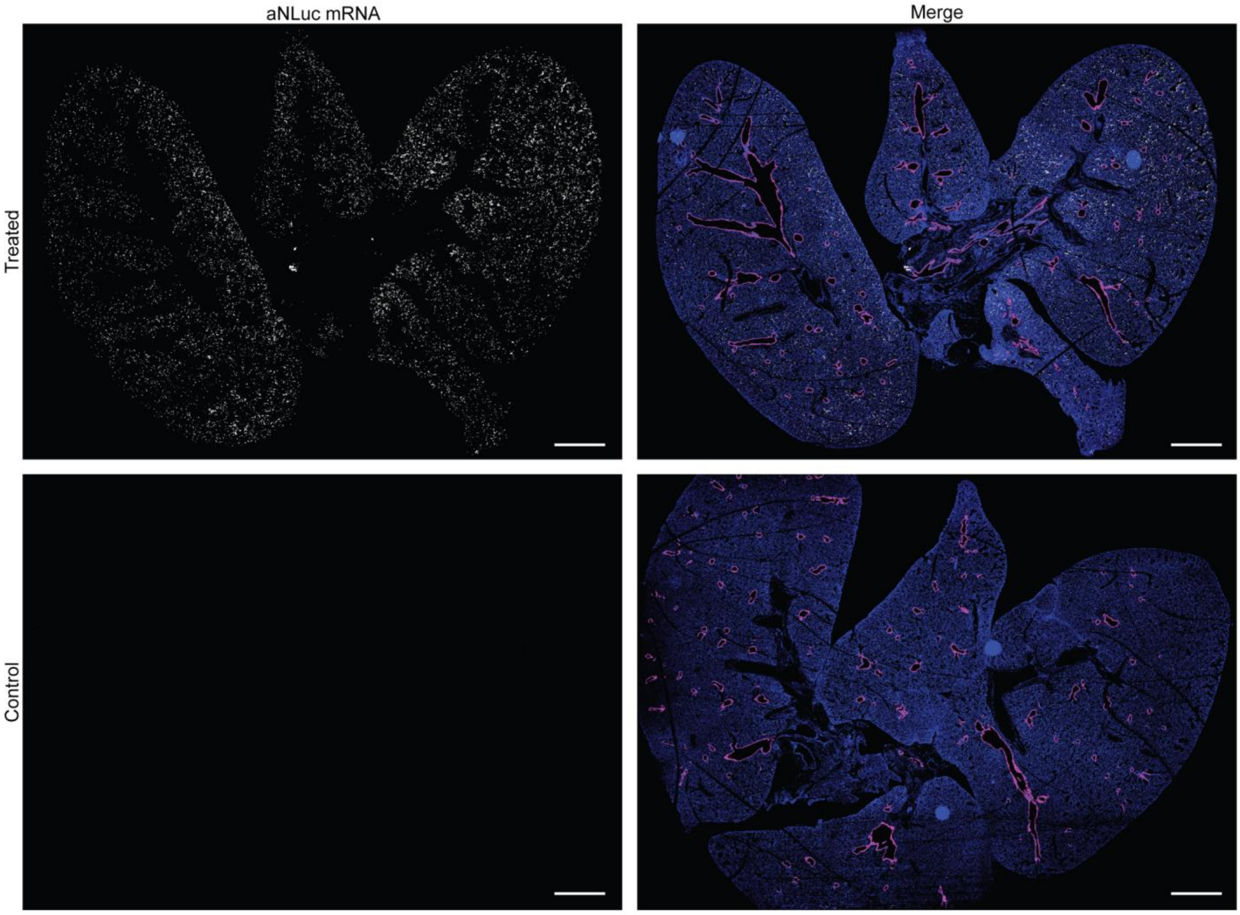

Whole lung images of hamster lungs from Fig. 3E. Nonciliated bronchiolar cells (magenta) and transfected mRNA (white) were localized using ISH for CC10 or aNLuc, respectively. Scale bar represents 2 mm.

Figure S4.

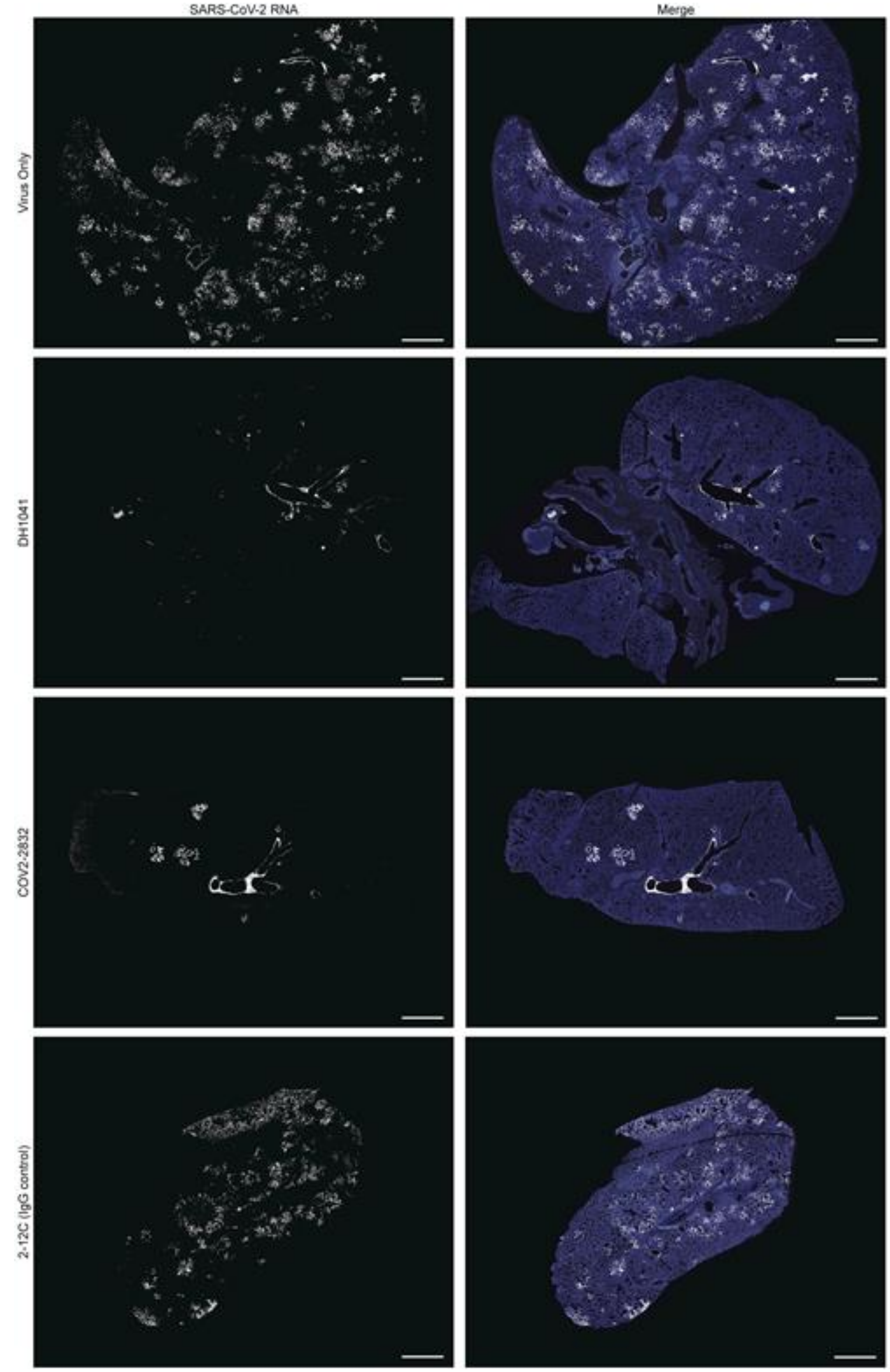

Whole lung images of hamster lungs from Fig. 3F were analyzed by ISH for club cell (magenta) and SARS-CoV-2 N (white) RNA localization. Scale bar represents 2 mm.
